# Change Point Analysis for Detecting Vaccine Safety Signals

**DOI:** 10.3390/vaccines9030206

**Published:** 2021-03-02

**Authors:** Seung-Hun You, Eun Jin Jang, Myo-Song Kim, Min-Taek Lee, Ye-Jin Kang, Jae-Eun Lee, Joo-Hyeon Eom, Sun-Young Jung

**Affiliations:** 1 College of Pharmacy, Chung-Ang University, Seoul 06974, Korea; dbtmdgnssla@cau.ac.kr (S.-H.Y.); myosong@cau.ac.kr (M.-S.K.); come410@cau.ac.kr (M.-T.L.); asnmoty11000@cau.ac.kr (Y.-J.K.); lje427@cau.ac.kr (J.-E.L.); eomjoohyeon@cau.ac.kr (J.-H.E.); 2 Department of Global Innovative Drugs, Graduate School of Chung-Ang University, Seoul 06974, Korea; 3 Department of Information Statistics, Andong National University, Andong 36729, Korea; ejjang@anu.ac.kr

**Keywords:** change point analysis, vaccines, data mining, pharmacovigilance, adverse events, signal detection, human papilloma virus vaccines

## Abstract

It is important to detect signals of abrupt changes in adverse event reporting in order to notice public safety concerns and take prompt action, especially for vaccines under national immunization programs. In this study, we assessed the applicability of change point analysis (CPA) for signal detection in vaccine safety surveillance. The performances of three CPA methods, namely Bayesian change point analysis, Taylor’s change point analysis (Taylor-CPA), and environmental time series change point detection (EnvCpt), were assessed via simulated data with assumptions for the baseline number of events and degrees of change. The analysis was validated using the Korea Adverse Event Reporting System (KAERS) database. In the simulation study, the Taylor-CPA method exhibited better results for the detection of a change point (accuracy of 96% to 100%, sensitivity of 7% to 100%, specificity of 98% to 100%, positive predictive value of 25% to 85%, negative predictive value of 96% to 100%, and balanced accuracy of 53% to 100%) than the other two CPA methods. When the CPA methods were applied to reports of syncope or dizziness following human papillomavirus (HPV) immunization in the KAERS database, Taylor-CPA and EnvCpt detected a change point (Q2/2013), which was consistent with actual public safety concerns. CPA can be applied as an efficient tool for the early detection of vaccine safety signals.

## 1. Introduction

Vaccines are generally administered to healthy individuals and a high standard of safety is therefore expected for vaccines. Furthermore, it is essential to maintain public confidence through post-licensure vaccine safety monitoring, as clinical trials might not have a large enough sample size to detect rare adverse events (AEs), which may occur in large post-licensure populations [1,2,3]. An additional feature of post-licensure vaccine safety monitoring is that it requires timely assessment in order to help distinguish true vaccine adverse reactions from coincidental unrelated events, particularly as potential health risks associated with vaccines are drawing increasing public attention [1]. Especially in the recent situation of rapid development of coronavirus disease 2019 (COVID-19) vaccines [4], early detection of both true safety signal and increased public concerns due to misinformation is important, to achieve adequate vaccine effectiveness, safety, and acceptance.

To detect signals of vaccine-related AEs from spontaneous reporting, data mining techniques based on finding disproportionalities through the proportional reporting ratio (PRR) [5], reporting odds ratio (ROR) [6], or the information component of the Bayesian confidence propagation neural network [7] have been widely used. However, because disproportionality analysis is an approach that analyzes AEs for a vaccine of interest in comparison to the same event for all other vaccines, the method may not be sufficient for detecting abrupt increases in safety reports. To enable early response, including epidemic investigations, causality assessment, and appropriate decision-making, early detection of a cluster of AEs following vaccination is important [8]. 

Change point analysis (CPA) is a methodology for detecting changes within a given time series or sequence. The CPA method focuses on detecting changes within time-series data and has been applied in climatology and medical imaging fields [9]. Several CPA methods have been developed using nonparametric, frequentist, or Bayesian approaches [10,11,12].

In the field of public health surveillance, with the exception of the application of CPA for active syndromic surveillance of emergency visits due to daily influenza-like illness during the H1N1 pandemic [13] and with regard to the safety issue of an appetite suppressant drug [14], vaccine safety applications are lacking. As vaccine safety issues are associated with distinguished features, an assessment of the applicability of CPA for vaccine safety is needed. To assess the validity of CPA in vaccine safety, the safety issues in human papillomavirus (HPV) vaccines can be considered. The HPV vaccine was first approved in 2006 to prevent cervical cancer and is included in national immunization programs (NIPs) worldwide [15,16,17,18]. In Japan, in March 2013, two syndromes, complex regional pain syndrome (CRPS) and postural orthostatic tachycardia syndrome (POTS) were reported in girls who received HPV vaccines, which drew public attention worldwide [17]. However, the Medicines and Healthcare Products Regulatory Agency provided evidence that there is no association between HPV vaccination and CRPS in August 2013 [19,20]. In addition, the European Medicines Agency (EMA) reported a lack of sufficient evidence for causality between HPV vaccinations and the two syndromes [16]. 

This study aimed to assess the applicability of various CPA methods for signal detection in vaccine safety surveillance. We considered Taylor’s change point analysis (Taylor-CPA), a method based on the nonparametric approach; environmental time series change point detection (EnvCpt) [21], based on the frequentist method; and the Bayesian change point (BCP) method [22].We first generated simulated data based on the framework of the real data reported to the Korea Adverse Event Reporting System (KAERS) in order to assess the performance of CPA. The methods were then applied to data regarding safety concerns of dizziness or syncope following HPV vaccination in the KAERS database for signal detection.

## 2. Materials and Methods

### 2.1. Data Source

Vaccine-related AE reporting data in this study were obtained from the spontaneous individual case safety reports (ICSRs) reported in the KAERS of the Korea Institute of Drug Safety and Risk Management. The KAERS database is composed of eight distributed tables including general information, information regarding the administered drug, AE information, serious adverse drug reaction cases, reporter information, causality assessment information of the drug–AE combination, patient medical history, and the sequence of reporting, such as initial and follow-up reports [23]. All vaccine names were coded using the Anatomical Therapeutic Chemical (ATC) classification system and AEs were coded using the WHO Adverse Reaction Terminology (WHO-ART), version 092 [24]. WHO-ART is a dictionary meant to serve as a basis for rational coding of adverse reaction terms in several countries and has four hierarchical structures: system-organ class (SOC), high-level term (HLT), preferred term (PT), and included term (IT). The study protocol was exempted from review by the Institutional Review Board of Chung-Ang University (IRB number: 1041078-201903-HR-071-01).

### 2.2. Study Vaccine and Adverse Event

To apply CPA methods to actual reported data, we analyzed ICSRs for the HPV vaccine (ATC code: J07BM) in the KAERS database from 2008 to 2014. Among these reports, AEs of interest were syncope and dizziness, which are known to be the leading symptoms of POTS [25].

### 2.3. Statistical Analysis

#### 2.3.1. Change Point Analysis Method

The Taylor-CPA method, which detects changes in time-series data in a nonparametric manner, iteratively performs the procedure that calculates the cumulative sum and bootstrapping methods without assuming parameters [10]. The EnvCpt method estimates the change point (CP) using the maximum likelihood estimation method and selects the best model as the one with the smallest Akaike information criterion [9,12,26]. The BCP method assumes a Bayesian model with a normal likelihood and estimates the posterior probability of the CP being at each location using the Markov chain Monte Carlo method [11]. In Bayesian methods, the probabilities for the hypotheses of interest can be directly represented using the posterior probabilities [27]. Since the posterior probability of the CP represents the probability that each point will be a change point, we defined it as a change point if the posterior probability of being a change point is greater than 90%. The threshold probability of 90% was selected based on previous studies [28,29,30].

#### 2.3.2. Simulation Study 

We considered various baseline values for the number of reports and the degree of change to reflect spontaneous ICSRs. The baseline number of reports was determined based on the mean number of reports in the KAERS database for known common AEs, such as injection site reactions, fever, and allergic reactions, and rare AEs, such as Guillain–Barré syndrome (GBS) [31].

**Figure 1:**
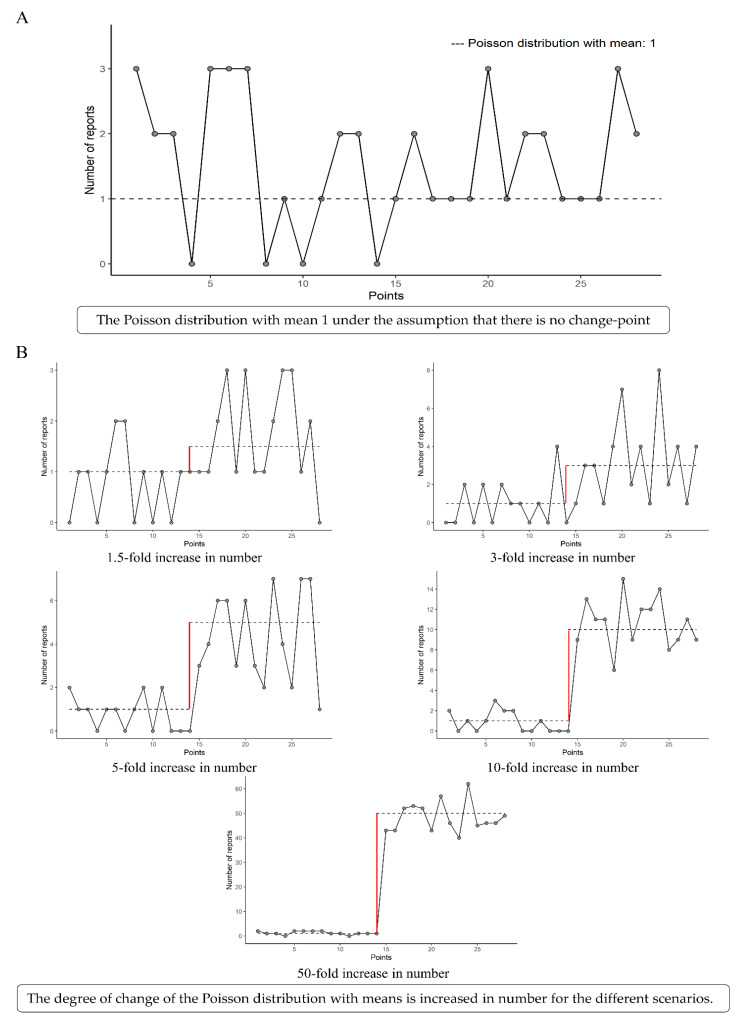
Simulation model from the Poisson distribution with a mean baseline number of reports of 1 (**A**) and the different scenarios with 1.5-, 3-, 5-, 10-, and 50-fold increases in number (**B**).

To implement the framework of baselines in which the number of common and rare AEs was reported, we generated 28 observations of baseline data from the Poisson distributions with means of 1, 5, 10, 50, and 100 (Figure 1A). We assumed that the change point was the midpoint (the 14th point) and that the degrees of change were 1.5-, 3-, 5-, 10-, and 50-fold increases at baseline (Figure 1B). Therefore, we generated 1000 datasets for each of the 25 scenes of the simulation scenarios, which combined the baseline and the degree of change. Three methodologies, namely Taylor-CPA, BCP, and EnvCpt, were applied to each scene.

In all scenes, we assumed that a significant change occurred at the 15th point of the 28 points. By allowing one point of margin, we defined the gold standard of classified CPs as the 14th, 15th, and 16th points out of the 28 points, and the remaining 25 points were treated as false CPs. To assess the performance for CPs detected by each CPA method, we constructed a 2*2 confusion matrix which showed the number of correctly and incorrectly classified conditions (Table S1) [32] and calculated accuracy (proportion of correctly classified observations), sensitivity (proportion of positive cases correctly predicted), specificity (proportion of negative cases correctly predicted), positive predictive value (PPV, proportion of true positives in the total positive predictions), negative predictive value (NPV, proportion of true negatives in the total negative predictions), and balanced accuracy (arithmetic means of sensitivity and specificity) (Table S2) [32,33].

#### 2.3.3. Application to the KAERS Database 

We applied three CPA methods to actual reports of syncope and dizziness following HPV vaccination in the KAERS database between 2008 and 2014 and compared the CPs detected with the three CPA methods. 

All analyses were performed using SAS 9.4 for Windows (SAS Institute, Inc., Cary, NC, USA) and R Statistical Software (version 4.0.0; R Foundation for Statistical Computing, Vienna, Austria).

## 3. Results

### 3.1. Performance Assessment for the Simulation Study

For all baselines with 1.5- and 3-fold increases, the three methods performed with accuracies from 95% to 100%, balanced accuracies from 50% to 100%, and PPVs and NPVs from 8% to 100% and 96% to 100%, respectively (Table 1, Table S3). When five- and tenfold increases were reported, the three methods performed with accuracies ranging from 97% to 100%, balanced accuracies from 63% to 100%, and PPVs and NPVs from 68% to 100% and 97% to 100%, respectively (Table 1, Table S3). When a 50-fold degree-of-change increase was reported, the accuracy increased from 99% to 100%, the balanced accuracy increased from 99% to 100%, and the PPV and NPV increased from 79% to 98% and 100%, respectively (Table 1, Table S3). For the results of applying the radar chart to visualize the 25 scenes of scenario simulation, the Taylor-CPA method was primarily the highest rank, and therefore widely visually distributed. The method was the highest especially for most metrics in the scenes that were combined with the baselines of 1, 5, and 10 and with degrees of change of 1.5- and 3-fold increases (Figure S1).

### 3.2. CPA for KAERS Database

Among the ICSRs in the KAERS database from 1988 to 2014, 2468 ICSRs were associated with the HPV vaccine. After excluding the follow-up reports and reporting errors, 2229 ICSRs for the HPV vaccine were identified (Figure S2). Among these, 155 ICSRs related to the AEs of interest.

**Figure 2:**
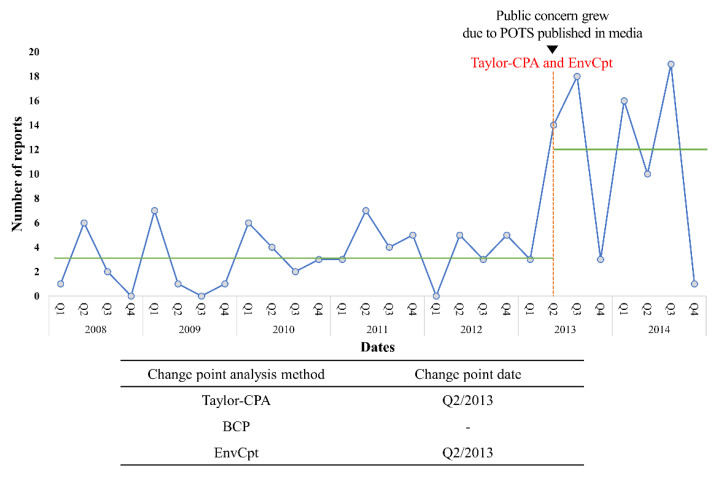
Change points detected by change point analysis based on the number of reports for the human papillomavirus vaccine including syncope and dizziness in individual case safety reports. Abbreviations: POTS, postural orthostatic tachycardia syndrome; BCP, Bayesian change point; Taylor-CPA, Taylor’s change point analysis; EnvCpt, environmental time series change point detection.

Among the three CPA methods, Taylor-CPA and EnvCpt detected the same significant CP from ICSRs of dizziness or syncope following HPV vaccination. The CP that was detected with the two methods was the same point in the second quarter of 2013 and, on average, 3.2 cases per quarter were reported until the CP, after which this figure soared to 12 (Figure 2). This point was consistent with a safety concern regarding dizziness or syncope following the HPV vaccine.

## 4. Discussion

In the present study, we assessed the applicability of CPA in detecting the signal of an abrupt increase in AE reports in vaccine safety surveillance. We examined the performances of three CPA methods, namely Taylor-CPA, BCP, and EnvCpt, using simulations based on the framework of the KAERS. We then applied the CPA to actual reports of syncope and dizziness following HPV vaccination in the KAERS database for actual public safety concern detection.

In the simulation study of 25 scenes with combined baseline numbers and degrees of changes, the Taylor-CPA method showed higher performance in terms of the overall assessment of the six indices. This result showing the higher robustness of the Taylor-CPA method compared with the BCP method is consistent with a previous study of surveillance of daily influenza-like illness emergency department visits [13]. Whereas the previous study qualitatively compared detected CPs, our study had a strength in that we compared the performances of three CPA methods by calculating reliability indices. 

The utilization of actual patterns of safety reports to generate a simulation framework is another strength of the present study. To determine the baseline number of reports before a subtle change, we performed a descriptive analysis of ICSRs reported in the KAERS database. The mean number of reports for known common AEs was 200 for injection site reactions, 90 for fever, and 10 for allergic reactions [31]. The mean number of ICSRs for known rare AEs, such as neuritis (including GBS), was two. Therefore, we defined 1, 5, 10, 50, and 100 reports and generated scenarios using a Poisson distribution. In the case of reports on syncope or dizziness for HPV vaccines, the degree of change in Q2 2013 was fivefold. As three or more reports are generally assumed to be significant in the pharmacovigilance field, we defined 1.5-, 3-, 5-, 10-, and 50-fold increases in reports as the possible degrees of change in the simulation models. 

Although the Taylor-CPA method assured 80% to 100% balanced accuracy when there was a threefold or more increase in report numbers, in the case of a 1.5-fold increase, scenes with less than 10 baseline reports showed a balanced accuracy of 67% and 53%. In particular, the scene of the one-report baseline with a 1.5-fold increase showed lower sensitivity (7%). Based on our simulation study, it should be noted that, when CPA methods are applied in practice, monitoring of rare events needs to be done cautiously because the methods may be underpowered. 

When we applied the three CPA methods to a nationwide spontaneous AE database, the KAERS database, the Taylor-CPA and EnvCpt methods detected the point of an actual public safety concern regarding syncope and dizziness after HPV vaccination. The second quarter of 2013 was consistent with the time the case related to POTS after HPV vaccination was publicized through media and newspaper articles in Japan. Soon, public concerns were raised worldwide through social media, for instance rejecting the safety of the HPV vaccine [20,34]. Spontaneous reporting of AEs may be stimulated by the behavioral influence of media publicity [35], for which prompt action for causality assessment is important, especially for vaccines under national immunization programs. Therefore, CPA can be applied as an efficient tool for the early detection of clusters of AE reports.

In the present study, we applied the Taylor-CPA method to the number of AE reports. In a previous study using the French pharmacovigilance database and EudraVigilance to detect a signal with regard to aortic valve incompetence (AVI) associated with the use of benfluorex, the CPA method was applied to not only the number of reports but also the proportional reporting ratio (PRR). In our database, our analysis was applied to the lower bound of the PRR and the percentage of reports did not show a significant change point. This difference may have been due to the nature of AEs. Our study detected signals resulting from increases associated with external factors, such as public service advertisements or national systems, rather than increases related to AEs. However, the use of benfluorex is associated with a significant increase in AVI [14]. Nevertheless, in the French National Agency for Medicines and Health Products Safety, the study examined the advantages of detecting initial signals and reducing misclassification by combining CPA analysis and the lower bound of PRR in pharmacovigilance [36]. Further research is needed to understand how different reasons depend on different measures, including the number of AE reports, the lower bound of the PRR, and the percentage of reports. 

Timeliness in detecting a CP is important to ensure prompt response, including further investigations and causality assessment. In our simulation analysis, we allowed one point of margin for setting the gold standard of the CPs. On the other hand, an Australian study based on weekly analysis of AEs reported that they aimed to detect a vaccine safety signal within three weeks [30]. Further research comparing CPA methods in respect of the timeliness of detecting a CP and comparing the degree of the margin used for CPs would be meaningful.

In the present study, we assumed a single point of abrupt increase as the CP because it is important to detect the exact point of change in pharmacovigilance. However, multiple points can be specified if the objective is to identify multiple points. In addition, although we performed univariate analyses for the three CPA methods, further research applying an algorithm using multivariate analysis would be possible. 

Our results need to be interpreted in light of several limitations inherent to spontaneous reporting systems. First, there may be problems of under-reporting and selective reporting, as only a minority of AEs are identified and reported. The heterogeneity of the original reporters may also affect the profiles of reported AEs. In the KAERS database, syncope or dizziness was more frequently reported by consumers and less frequently by healthcare professionals than other ICSRs following HPV vaccination (Table S4). Second, because the reports are made spontaneously by consumers and healthcare professionals, and causality assessment is not essential, the detected point of sharp increase in reports could not be interpreted as a causal relationship; the CP only implies a signal for safety issues that necessitates further investigation. In the case of POTS and CRPS following HPV vaccination, further assessments were made by regulatory authorities, which documented low relevance for the link between the HPV vaccine and the AEs [16]. 

Nevertheless, a spontaneous reporting database can provide an opportunity to monitor vaccine safety and identify new, rare signals to generate ideal prospective research hypotheses. Whereas traditional data-mining approaches are based on the disproportionality of reports, CPA may further contribute to earlier detection of vaccine safety concerns, especially for newly implemented vaccines, such as COVID-19 vaccines. In cases of detection of a CP, immediate investigation and causality assessment would be needed to provide appropriate safety information and to minimize vaccine refusal in the population which could diminish the efficacy of the vaccine. 

## 5. Conclusions

In our simulation study, the Taylor-CPA method exhibited the best performance for the detection of a change point compared to the other two CPA methods. Based on our application examples, the CPA could be used as an effective tool for the early detection of vaccine safety signals within a time series of spontaneous AE reporting systems.

## Figures and Tables

**Table 1:** Balanced accuracy of performance results obtained with the three change point analysis methods on the 1000 simulated datasets for 25 scenes.

Degree of Change	Mean Baseline Number of Reports														
	1			5			10			50			100		
	BCP	Taylor -CPA	EnvCpt	BCP	Taylor -CPA	EnvCpt	BCP	Taylor -CPA	EnvCpt	BCP	Taylor -CPA	EnvCpt	BCP	Taylor -CPA	EnvCpt
The number of reports increased															
1.5-fold	50%	53%	52%	50%	67%	56%	52%	80%	69%	75%	98%	99%	93%	99%	100%
3-fold	52%	80%	73%	77%	98%	99%	93%	99%	100%	100%	100%	100%	100%	100%	100%
5-fold	63%	95%	95%	97%	100%	100%	100%	100%	100%	100%	100%	100%	100%	100%	100%
10-fold	90%	99%	99%	100%	100%	100%	100%	100%	100%	100%	100%	100%	100%	100%	100%
50-fold	99%	100%	99%	100%	100%	100%	100%	100%	100%	100%	100%	100%	100%	100%	100%

Abbreviations: BCP, Bayesian change point; Taylor-CPA, Taylor’s change point analysis; EnvCpt, environmental time series change point detection; PPV, positive predictive value; NPV, negative predictive value.

## Data Availability

Restrictions apply to the availability of these data. Data was obtained from Korea Institute of Drug Safety and Risk Management (KIDS) and are available https://open.drugsafe.or.kr/ (accessed on 12 February 2021) with the permission of KIDS.

## Supplementary Materials

The following are available online at https://www.mdpi.com/2076-393X/9/3/206/s1. Figure S1: Data visualization with a radar chart was defined (A) and 25 scenes of the simulation were visualized, combining the mean number of reports and the degree of change using six metrics of the confusion matrix (B): accuracy; sensitivity; specificity; positive predictive value; negative predictive value; and balanced accuracy. Figure S2: Flow diagram of individual case safety reports. Table S1: The confusion matrix for a simulation model: possible results from a binary classier. Table S2: Statistics for performance assessment derived from the confusion matrix. Table S3: Summary of performance results obtained with the three change point analysis methods for the 1000 simulated datasets for 25 scenes. Table S4: Characteristics of individual case safety reports for the human papillomavirus vaccine and other vaccines.
