# Supplementary material for: Change Point Analysis for Detecting Vaccine Safety Signals
Source: Vaccines (Basel). 2021 Mar 2;9(3):206. doi: 10.3390/vaccines9030206 (PMC8001699; doi:10.3390/vaccines9030206)
Supplement: Supplementary file 1 [file vaccines-09-00206-s001.pdf]

vaccines

MDPI

Table S1. The confusion matrix for a simulation model: Possible results from a binary classier.

		**Condition determined by "Gold Standard"**	
		**Positive**	**Negative**
Classified condition	Positive	True Positive (TP)	False Positive (FP)
	Negative	False Negative (FN)	True Negative (TN)

Table S2. Statistics for performance assessment derived from the confusion matrix.

Metrics	Formula	Definition
Accuracy	$\frac{TP + TN}{TP + FP + FN + TN}$	The proportion of correctly classified observations
Sensitivity	$\frac{TP}{TP + FN}$	The proportion of positive cases correctly predicted
Specificity	$\frac{TN}{FP + TN}$	The proportion of negative cases correctly predicted
PPV	$\frac{TP}{TP + FP}$	The proportion of true positive in the total of positive predictions
NPV	$\frac{TN}{FN + TN}$	The proportion of true negatives in the total of negative predictions
Balanced accuracy	$\frac{\text{Sensitivity} + \text{Specificity}}{2}$	The arithmetic means of the two metrics (sensitivity and specificity), that is the highest powerful and useful when the classes imbalanced.

Abbreviations: PPV, Positive predicted value; NPV, Negative predicted value; TP, True Positive; FP, False Positive; FN, False Negative; TN, True Negative.

Table S3. Summary of performance results obtained with the three change point analysis methods on the 1,000 simulated data for 25 scenes.

	Mean baseline number of reports														
	1			5			10			50			100		
	BCP	Taylor -CPA	Env Cpt	BCP	Taylor -CPA	Env Cpt	BCP	Taylor -CPA	Env Cpt	BCP	Taylor -CPA	Env Cpt	BCP	Taylor -CPA	Env Cpt
1.5-fold increase in number															
accuracy	96%	96%	95%	96%	96%	96%	96%	97%	97%	98%	99%	100%	99%	99%	100%
sensitivity	1%	7%	6%	1%	35%	13%	3%	62%	38%	49%	97%	98%	85%	100%	100%
specificity	100%	99%	99%	100%	99%	99%	100%	98%	99%	100%	99%	100%	100%	99%	100%
PPV	8%	25%	19%	39%	48%	44%	67%	61%	66%	99%	82%	96%	100%	84%	98%
NPV	96%	96%	96%	96%	97%	97%	96%	98%	98%	98%	100%	100%	99%	100%	100%
balanced accuracy	50%	53%	52%	50%	67%	56%	52%	80%	69%	75%	98%	99%	93%	99%	100%
3-fold increase in number															
accuracy	96%	97%	97%	98%	99%	100%	99%	99%	100%	100%	99%	100%	100%	99%	100%
sensitivity	5%	61%	47%	53%	97%	98%	87%	100%	100%	100%	100%	100%	100%	100%	100%
specificity	100%	98%	99%	100%	99%	100%	100%	99%	100%	100%	99%	100%	100%	99%	100%
PPV	34%	61%	59%	96%	82%	96%	98%	84%	97%	100%	84%	98%	100%	85%	98%
NPV	96%	98%	98%	98%	100%	100%	99%	100%	100%	100%	100%	100%	100%	100%	100%
balanced accuracy	52%	80%	73%	77%	98%	99%	93%	99%	100%	100%	100%	100%	100%	100%	100%
5-fold increase in number															
accuracy	97%	99%	99%	100%	99%	100%	100%	99%	100%	100%	99%	100%	100%	99%	100%
sensitivity	27%	91%	90%	95%	100%	100%	100%	100%	100%	100%	100%	100%	100%	100%	100%
specificity	99%	99%	99%	100%	99%	100%	100%	99%	100%	100%	99%	100%	100%	99%	100%
PPV	68%	78%	84%	96%	84%	98%	97%	83%	97%	99%	83%	98%	100%	85%	98%
NPV	97%	100%	100%	100%	100%	100%	100%	100%	100%	100%	100%	100%	100%	100%	100%
balanced accuracy	63%	95%	95%	97%	100%	100%	100%	100%	100%	100%	100%	100%	100%	100%	100%
10-fold increase in number															
accuracy	98%	99%	100%	100%	99%	100%	100%	99%	100%	100%	99%	100%	100%	99%	100%
sensitivity	81%	99%	98%	100%	100%	100%	100%	100%	100%	100%	100%	100%	100%	100%	100%
specificity	99%	99%	100%	100%	99%	100%	100%	99%	100%	100%	99%	100%	100%	99%	100%
PPV	80%	84%	90%	93%	84%	98%	96%	84%	97%	99%	84%	98%	99%	85%	98%
NPV	99%	100%	100%	100%	100%	100%	100%	100%	100%	100%	100%	100%	100%	100%	100%
balanced accuracy	90%	99%	99%	100%	100%	100%	100%	100%	100%	100%	100%	100%	100%	100%	100%
50-fold increase in number															
accuracy	99%	99%	99%	100%	99%	100%	100%	99%	100%	100%	99%	100%	100%	99%	100%
sensitivity	100%	100%	98%	100%	100%	100%	100%	100%	100%	100%	100%	100%	100%	100%	100%
specificity	99%	99%	100%	100%	99%	100%	100%	99%	100%	100%	99%	100%	100%	99%	100%
PPV	79%	84%	90%	92%	85%	98%	95%	83%	97%	98%	84%	98%	98%	85%	98%
NPV	100%	100%	100%	100%	100%	100%	100%	100%	100%	100%	100%	100%	100%	100%	100%
balanced accuracy	99%	100%	99%	100%	100%	100%	100%	100%	100%	100%	100%	100%	100%	100%	100%

Abbreviations: BCP, the Bayesian change point; Taylor-CPA, Taylor's change point analysis; EnvCpt, the environmental time series change point detection; PPV, positive predictive value; NPV, negative predictive value.

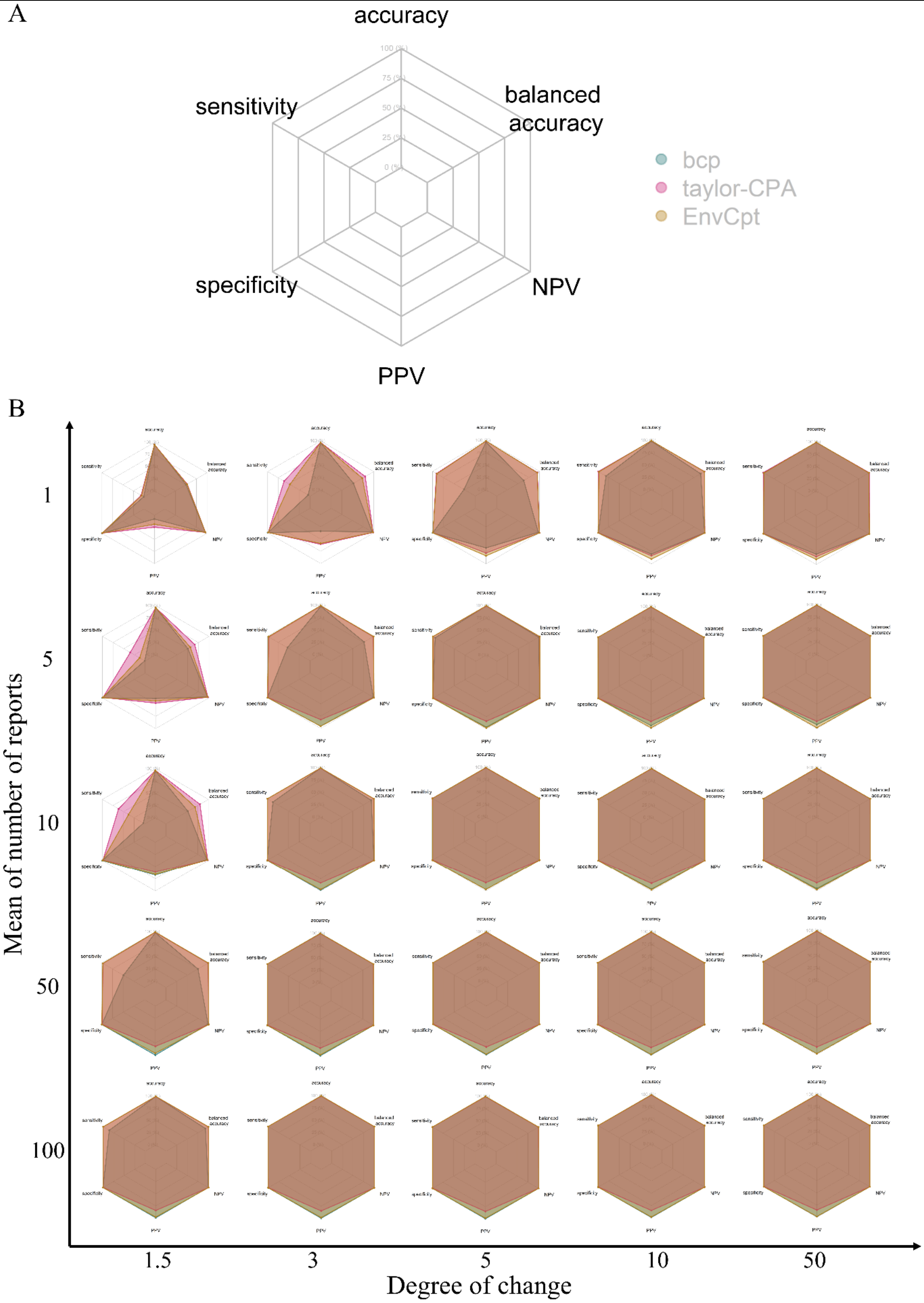

Figure S1. Data visualization with a radar chart is defined (A) and 25 scenes of the simulation are visualized combined mean of the reports and degree of change using 6 metrics of the confusion matrix (B): accuracy; sensitivity; specificity; positive predicted value; negative predicted value; balanced accuracy.

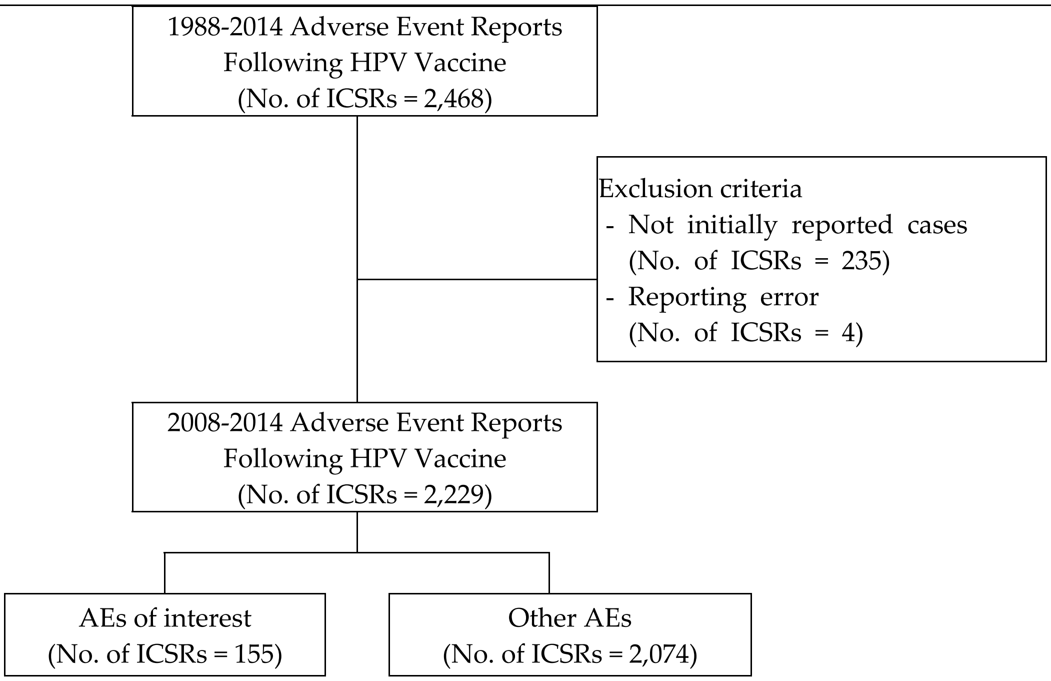

Figure S2. Flow diagram of individual case safety reports. Abbreviations: ICSRs, Individual Case Safety Reports; AEs, Adverse Events; HPV, human papillomavirus.

vaccines

MDPI

Table S4. Characteristics of individual case safety reports of syncope or dizziness and other events following human papillomavirus vaccine.

Characteristics	Total reports (*N* = 2,229) *N*	(%)	Reports of syncope or dizziness (*N* = 155) *N*	(%)	Other AEs (*N* = 2074) *N*	(%)	*p*-value
Sex							0.4341
Male	22	(1)	0	(0)	22	(1.1)	
Female	2,133	(95.7)	150	(96.8)	1,983	(95.6)	
Unknown	74	(3.3)	5	(3.2)	69	(3.3)	
Age							0.0027
24 months - 11 years old	30	(1.3)	0	(0)	30	(1.4)	
12 - 18 years old	201	(9)	20	(12.9)	181	(8.7)	
19 - 64 years old	1,006	(45.1)	85	(54.8)	921	(44.4)	
Unknown	992	(44.5)	50	(32.3)	942	(45.4)	
Year report was received							<0.0001
2008	32	(1.4)	10	(6.5)	22	(1.1)	
2009	55	(2.5)	7	(4.5)	48	(2.3)	
2010	116	(5.2)	13	(8.4)	103	(5.)	
2011	200	(9.)	24	(15.5)	176	(8.5)	
2012	126	(5.7)	6	(3.9)	120	(5.8)	
2013	1,061	(47.6)	47	(30.3)	1,014	(48.9)	
2014	639	(28.7)	48	(31)	591	(28.5)	
Report Type							<0.0001
Spontaneous report	1,129	(50.7)	116	(74.8)	1,013	(48.8)	
Research (including PMS)	1,081	(48.5)	34	(21.9)	1,047	(50.5)	
Literature	1	(0)	0	(0)	1	(0)	
Others	18	(0.8)	5	(3.2)	13	(0.6)	
Original reporter							<0.0001
Healthcare professionals	1,556	(69.8)	89	(57.4)	1,467	(70.7)	
Consumers	332	(14.9)	27	(17.4)	305	(14.7)	
Others	204	(9.2)	21	(13.5)	183	(8.8)	
Unknown	137	(6.1)	18	(11.6)	119	(5.7)	
Reporter							0.0006
Regional PV centers	28	(1.3)	8	(5.2)	20	(1.)	
Manufacturer	2,073	(93.0)	137	(88.4)	1,936	(93.3)	
Medical institution	2	(0.1)	0	(0)	2	(0.1)	
Pharmacy	1	(0)	0	(0)	1	(0)	
Consumer	120	(5.4)	10	(6.5)	110	(5.3)	
Others	5	(0.2)	0	(0)	5	(0.2)	
Serious adverse event							<0.0001
Yes	135	(6.1)	34	(21.9)	101	(4.9)	
No	2,094	(93.9)	121	(78.1)	1,973	(95.1)	
Serious adverse event category							
Disability	3	(2.2)	0	(0)	3	(3)	0.5718
Life threatening	1	(0.7)	0	(0)	1	(1)	0.7481
Hospitalization	47	(34.8)	9	(26.5)	38	(37.6)	0.2377
Other medical events	91	(67.4)	28	(82.4)	63	(62.4)	0.0316

vaccines

MDPI

Abbreviations: AE, adverse events; HPV, human papillomavirus; PMS, post marketing surveillance; PV, pharmacovigilance.
